# Earthworm *Lumbricus rubellus* MT-2: Metal Binding and Protein Folding of a True Cadmium-MT

**DOI:** 10.3390/ijms17010065

**Published:** 2016-01-05

**Authors:** Gregory R. Kowald, Stephen R. Stürzenbaum, Claudia A. Blindauer

**Affiliations:** 1 Department of Chemistry, University of Warwick, Coventry CV4 7AL, UK; GKowald@acciuk.co.uk; 2 MRC-PHE Centre for Environment & Health, King´s College London, London SE1 9NH, UK; stephen.sturzenbaum@kcl.ac.uk; 3 Analytical and Environmental Sciences Division, Faculty of Life Science & Medicine, King’s College London, London SE1 9NH, UK

**Keywords:** metallothionein, cadmium, zinc, selectivity, protein folding, mass spectrometry, NMR spectroscopy

## Abstract

Earthworms express, as most animals, metallothioneins (MTs)—small, cysteine-rich proteins that bind d^10^ metal ions (Zn(II), Cd(II), or Cu(I)) in clusters. Three MT homologues are known for *Lumbricus rubellus*, the common red earthworm, one of which, wMT-2, is strongly induced by exposure of worms to cadmium. This study concerns composition, metal binding affinity and metal-dependent protein folding of wMT-2 expressed recombinantly and purified in the presence of Cd(II) and Zn(II). Crucially, whilst a single Cd_7_wMT-2 species was isolated from wMT-2-expressing *E. coli* cultures supplemented with Cd(II), expressions in the presence of Zn(II) yielded mixtures. The average affinities of wMT-2 determined for either Cd(II) or Zn(II) are both within normal ranges for MTs; hence, differential behaviour cannot be explained on the basis of overall affinity. Therefore, the protein folding properties of Cd- and Zn-wMT-2 were compared by ^1^H NMR spectroscopy. This comparison revealed that the protein fold is better defined in the presence of cadmium than in the presence of zinc. These differences in folding and dynamics may be at the root of the differential behaviour of the cadmium- and zinc-bound protein *in vitro*, and may ultimately also help in distinguishing zinc and cadmium in the earthworm *in vivo*.

## 1. Introduction

One of the most intriguing questions in the field of metal homeostasis concerns how biological systems distinguish and discriminate between different metal ions. This is important for understanding not only the metabolic pathways of essential metal ions, but also mechanisms of tolerance and detoxification including those for xenobiotic ions. As far as essential metal ions are concerned, it has been recognised that the cytosolic concentrations of essential metal ions are, in healthy conditions, regulated according to their relative position within the Irving-Williams series, and the proteins involved in their homeostasis typically have metal affinities to match these concentrations [1]. This ensures, for example, that Cu(I), the most competitive essential metal ion, is kept out of the binding sites of all other metal ions—even when, and this is frequently the case, the affinity of the respective protein is higher for copper than for the “correct” metal ion. In the case of a non-essential, toxic metal ion such as cadmium, it may be inferred that at least one protein with sufficiently high affinity is required, with the added proviso that this protein, once expressed, should ideally not significantly interfere with the metabolism of other metal ions. This becomes particularly important when the toxic and essential metal ions have relatively similar coordination chemistry, such as in the case of essential Zn(II) and toxic Cd(II). The current study will highlight that certain metallothioneins (MTs), small proteins with an extraordinarily high proportion of cysteine thiols that endows them with high affinity towards both Zn(II) and Cd(II), may display distinctly different behaviour towards these two closely related ions.

MTs were one of the first protein families to be associated with metal metabolism in animals [2,3,4]. Initially discovered in horse kidney cortex as major cadmium-binding proteins [5], their physiological functions in mammals are now thought by most workers in the field to be predominantly if not exclusively concerned with the metabolism of the essential zinc and copper metal ions. In addition, because of their high thiol content, they can also function as antioxidants, and may also link cellular redox status to zinc dynamics [6].

Unusually, and in contrast to most other protein families, MTs are a polyphyletic group of proteins held together not by significant similarities in protein sequence, but rather by a number of descriptors that refer to their overall composition (high proportion of sulfur and metals), and biophysical characteristics such as peculiar spectroscopic features indicative of metal–sulfur clusters. This fact should be reason enough to refrain from extrapolating the biological functions, structural features, or chemical reactivity of an MT from one phylum to another, but sadly, this is a frequent occurrence in the literature.

It has been pointed out that even though mammalian MTs undoubtedly bind cadmium *in vivo* and, hence, play a role in cadmium metabolism [7], it is unlikely that this action is a true, evolutionarily constrained function [8,9]. This may however be distinctly different for organisms in close contact with soils, *i.e.*, plants [10] and terrestrial invertebrates [11,12,13]. Typically, due to their relatively similar chemistries, toxic cadmium occurs in soils at concentrations that are only two to three orders of magnitude smaller than those of essential zinc. Moreover, in topsoils treated with rock phosphate fertilisers, cadmium levels can be significantly higher and reach up to 14 ppm [14]. With 2015 being the international year of soil [15], it seems appropriate to devote some attention to a group of very important soil organisms supremely adept at coping with cadmium: the earthworm.

Earthworms, master “soil engineers”, directly ingest and process soil. This does not only mean that they play a major role in the dynamics of nutrients and essential elements present in soil [16], but also that they are exposed to any compound present, both through their digestive tract as well as through their skin. Earthworms are therefore also used for biomonitoring purposes in ecotoxicology, and have also been dubbed “soil sentinels” [17].

At least some species, for example the common red earthworm *Lumbricus rubellus*, can survive in the presence of 600 µg cadmium per g dry weight of soil [18]. Intriguingly, *L. rubellus* also specifically bio-accumulates cadmium to a staggering ratio of up to 1 mg per gram dry body weight, whilst lead, zinc and copper are not bio-accumulated [18]. This difference clearly indicates that there must be distinct metabolic pathways for the chemically closely related Zn(II) and Cd(II). A similar conclusion has been drawn for other soil organisms; for example, the nematode *Caenorhabditis elegans* uses a system that includes phytochelatins, cystathionine, and its two metallothioneins MTL-1 and MTL-2 to discriminate between zinc and cadmium [19,20], with subsequent distinct pathways for utilisation and detoxification [21]. Given the differences in zinc and cadmium accumulation, it is conceivable that similar mechanisms may exist in earthworms, with a potential function for MTs in the discrimination between essential zinc and toxic cadmium.

**Figure 1:**
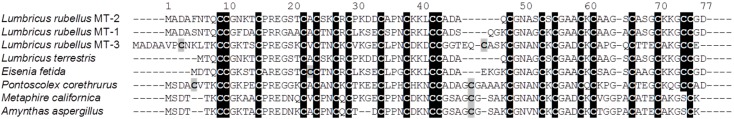
Sequence alignment of selected annelid MTs, showing conservation of two blocks of Cys-rich regions with 11–12 and 8–9 Cys residues (highlighted by black boxes). All three sequences from *L. rubellus* are shown; the numbering refers to wMT-2. Non-conserved Cys residues are highlighted in grey.

Many eukaryotic species, including invertebrates, express several different MT homologues (In MT literature, it is common to call these different forms “isoforms”, although strictly speaking this is a term that should refer to different forms derived from a single gene. However, in most if not all cases, different forms of MTs in one particular species are derived from different genes; therefore we prefer to refer to these as “homologues” provided that an evolutionary relationship between them is likely.), sometimes, but not always in a tissue-specific manner. Evidence has been accumulating that different homologues in the same species can exhibit different metal selectivities; prime examples are the Cu- and Cd-MTs from snails [22,23,24] as well as the *C. elegans* MTs mentioned above [19,20]. MTs are being studied in several annelids, mainly in terms of gene expression [25,26,27,28]. Perhaps the best-studied system in terms of MTs is *L. rubellus*; for this species, at least three MT genes are known [29]. The corresponding wMT-1 and wMT-2 proteins have been isolated from adult earthworms [18], whilst the protein sequence of a third homologue, wMT-3, has been derived from an EST library generated from developing cocoons [29] (Figure 1).

The protein sequences of wMT-1 and wMT-2 are 74.7% identical and 91.1% similar, whilst that of wMT-3 is only 56% identical and 67% similar to wMT-1 or wMT-2. The expression patterns of these three homologues also differ considerably; the *wMT-3* gene has been suggested to be highly expressed during embryonic development [29], whereas *wMT-1* and *wMT-2* are both responsive to metal exposure, but to different extents. *wMT-2* is the homologue with the most pronounced responsiveness to cadmium—its expression may be upregulated several hundred- to thousand-fold in response to high cadmium levels in soil [30]. The non-transience of this increased expression suggested that this constitutes the primary response to cadmium [31]. Accordingly, *wMT-2* was also one of the most upregulated genes in response to chronic cadmium exposure, as identified in an extensive transcriptomic study [32]. The mechanism for metallo-regulation of *wMT-2* genes—and indeed many other invertebrate MT genes—has puzzled researchers for some while, because even though three recognisable metal-response elements (MREs) are present in the pertinent upstream regions in all three identified *wMT-2* loci [29], a protein corresponding to MTF-1, the transcription factor that recognises MREs in vertebrates and *Drosophila melanogaster* [33], could not be identified. A recent study involving EMSA and DNAse I footprinting revealed that cytosolic, but not nuclear extracts from *L. rubellus* cells contain proteins capable of binding to the *wMT-2* promoter region in a zinc-dependent manner. The DNA footprinting experiments identified cAMP-responsive elements (CRE) as putative candidates for MT gene regulation in invertebrates [34].

The *wMT-2* gene is not only supremely responsive to cadmium exposure, its product was also the major Cd-binding protein isolated from earthworms from a contaminated site [18]. The highest levels of wMT-2 protein were found in Cd-exposed worms in the thyphlosole and gut epithelium (both alimentary surfaces), chloragogenous tissues (these have been likened to vertebrate livers), coelomocytes (a type of invertebrate immune cells) and nephridia (analogues of vertebrate kidneys) [35]. Thus, even though wMT-2, like any other MT, is also capable of binding other metal ions such as Zn(II) and Cu(I) [36], and even though gene expression may also be induced by metals other than cadmium [37], there are multiple lines of evidence, that as far as biological function is concerned, wMT-2 is a “cadmium-MT”. We have used a combination of mass spectrometry and NMR spectroscopy to study whether and how biological function may be reflected in the biophysical properties, in particular metal affinities and protein folding, of this invertebrate MT.

## 2. Results and Discussion

### 2.1. Production of Untagged Recombinant Zinc- and Cadmium-Bound wMT-2

Earthworm MT proteins have previously been studied *in vitro*; data for proteins isolated from their native host are available for *L. rubellus* [18] and *Eisenia fetida* [38], and recombinant, S-tagged wMT-2 has been studied as well [31,39].

**Figure 2:**
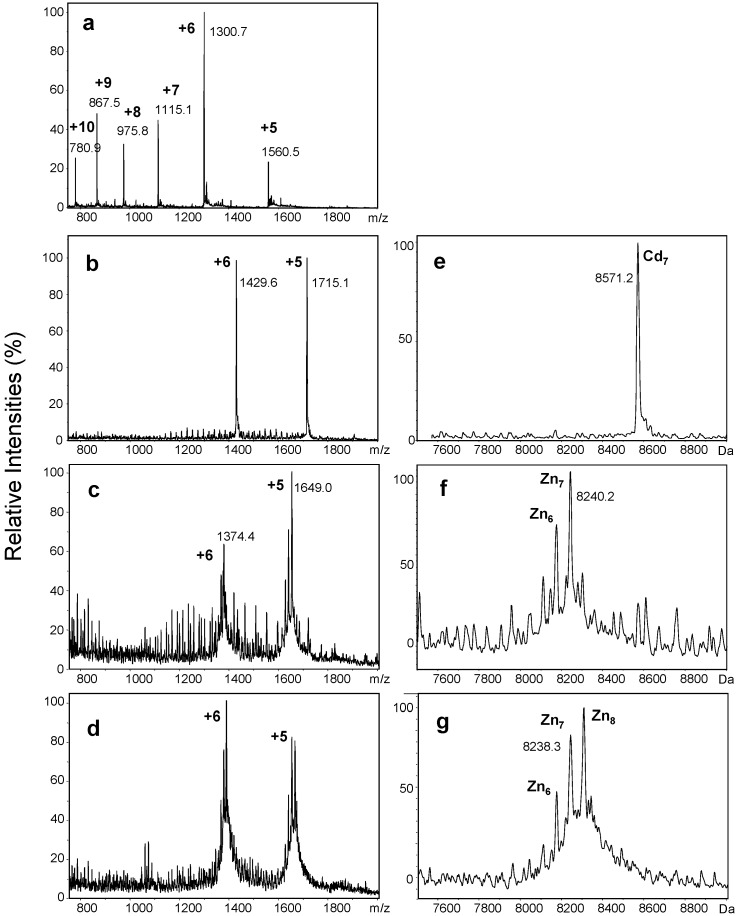
Raw (**a**–**d**) and deconvoluted (**e**–**g**) mass spectra of recombinant wMT-2 (10 mM NH_4_HCO_3_, 10% MeOH) (**a**) Raw mass spectrum showing charge states for demetallated wMT-2 at pH < 2.0. Deconvolution onto a true mass scale gives a neutral mass of 7798.4 Da; (**b**,**e**) MS data for wMT-2 expressed and purified in the presence of Cd(II); (**c**–**g**) MS data from two different expressions in the presence of Zn(II). Spectra (**b**–**f**) were recorded at pH 8.5, spectra (**d**,**g**) at pH 7.0. The low signal to noise ratio in spectra (**c**–**g**) is due to the low concentration of the protein, which in turn was due to rapid sample degradation. The series of additional peaks between *m*/*z* 1000 and 1700 seen in (**b**,**c**) correspond to polyethylene glycol, contamination with which can occur through use of ultracentrifugation filters.

In the present study, wMT-2 was also expressed recombinantly in *E. coli* as an S-tagged fusion protein. Although the S-tag is relatively short and is not expected to impact on metal-binding abilities, in the context of protein structure and folding, it is still preferable that the studied protein is as similar to the native form as possible. Therefore, the tag was removed by cleavage with thrombin, after a first purification step. Contrary to its intended purpose, the S-tag was not used to aid purification; instead, a three-step procedure involving gel filtration, thrombin cleavage, and a second round of gel filtration chromatography was found to give higher protein yields compared to attempts that included S-tag affinity-based purification. During expression, cultures were supplemented with either Zn(II) or Cd(II). The resulting purified proteins were analysed by electrospray ionisation mass spectrometry (Figure 2).

A mass of 7798.4 Da for the charge-neutral species, obtained under denaturing and hence demetallation conditions (pH < 2), agrees well with the theoretical mass of 7797.9 Da for apo-wMT-2, calculated from the sequence (UniProt entry O76955_LUMRU; Figure 1) plus the mass of an additional glycine and serine, which remain at the N-terminus after cleavage with thrombin. The low baseline and absence of significant peaks not belonging to the charge state series for apo-wMT-2 is an indication for the purity of this preparation. The mass spectral data shown in Figure 2b–g were obtained under “native” ESI conditions; this means that the proteins are brought into the gas phase in their folded, metallated forms, which can be achieved by working at non-acidic pH, and using no or only small quantities of organic solvent [40]. This approach is in principle applicable to non-covalent protein complexes including metalloproteins in general, but has proven outstandingly useful for the study of MTs, as it is the only method that allows distinction between different metallospecies [41,42,43,44,45,46]. Furthermore, MTs are particularly amenable to this analysis, as they are small proteins that ionise well. Only two charge states, +5 and +6, are observed for metallated wMT-2 irrespective of whether Zn(II)- or Cd(II)-bound (Figure 2b–d). A small number of charge states is indicative of a folded state [47], as not only are there a smaller number of protonatable groups exposed in a folded protein, but the latter is also more compact and, hence, can accommodate fewer charges of the same sign. Figure 2e shows the result of deconvolution of the data shown in Figure 2b onto a true mass scale. One single metallospecies—Cd_7_wMT-2 with a neutral mass of 8571.2 Da (theoretical: 8570.8 Da)—was observed for wMT-2 expressed in the presence of cadmium. A Cd_7_ species was also the major species previously observed for S-tagged Cd-wMT-2 at neutral pH [39]. Figures 2c–g demonstrate that MS data obtained for protein expressed in the presence of Zn(II) did not only present a lower signal-to-noise ratio, but that invariably, more than one metallospecies was observed. This was indeed also the case before cleavage of the S-tag (data not shown). Zn_7_ was a major species, but was accompanied by species with both higher and lower metal contents, depending on batch and sample pH. The significance of this observation will be discussed later on.

### 2.2. Proton-Driven Metal Loss

**Figure 3:**
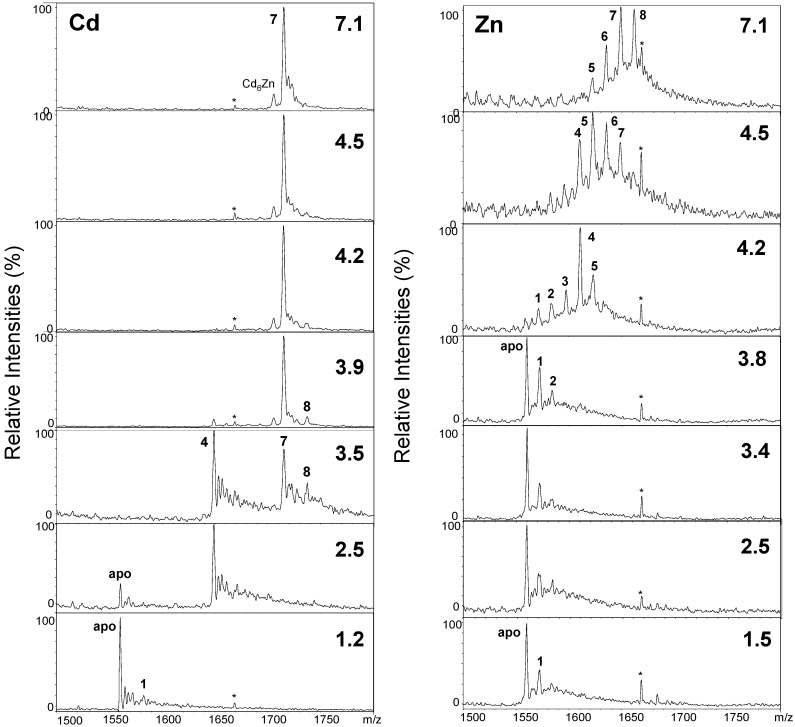
+5 charge state of raw, smoothed mass spectra showing speciation of Cd-wMT-2 and Zn-wMT-2 at different pH values (10 mM NH_4_HCO_3_, 10% MeOH). The peak labelled in both series with an asterisk is an unknown contaminant. The numbers indicate how many metal ions are bound in the respective species. All observed masses are compiled in Table 1.

A pH titration monitored by mass spectrometry was undertaken for both the zinc and cadmium forms of wMT-2 (Figure 3). Both zinc- and cadmium-loaded wMT-2 eventually lost their bound metals due to competition from protons for the thiolate sulfurs, but like every MT studied so far by this method, the cadmium-bound form exhibited substantially higher pH-stability, with the Cd_7_ species still dominant at pH 3.9, whilst the Zn(II)-containing preparation at similar pH was dominated by the apo-form. This lower stability of Zn-MTs *versus* Cd-MTs is expected, as Cd-S bonds are inherently stronger than Zn-S bonds [3]. From these data (note that the full dataset contained more datapoints), it was possible to estimate pH-of-half-displacement values (pH(1/2); also called apparent p*K*_a_ values) of 2.8 for Cd-wMT-2 and 4.2 for Zn-wMT-2. It should be pointed out that ESI-MS is not the method of choice for the determination of pH(1/2) values, as *m*/*z* peak intensities do not only depend on the concentration of the respective species in solution, but also their ionisation efficiency, which may or may not be comparable for different species [48], depending on their degree of (un-)folding. In the present case though, the value obtained for Cd-wMT-2 agreed within error limits with the pH(1/2) value of 2.9 previously determined via UV–Vis spectroscopy for S-tagged Cd-wMT-2 [31]. Both values for Cd- and Zn-wMT-2 are within ranges expected for MTs [3], with both being on the low side, which might be taken for an indication of higher stability—but only as far as competition with protons is concerned (as will be seen in Section 2.3).

As indicated before, ESI-MS is the only technique that is able to directly give detailed speciation information, and as such, can be used to explore cooperativity in the binding of multiple metals. The data shown in Figure 3 provide a classical example of cooperative (for Cd-wMT-2) *versus* largely non-cooperative (for Zn-wMT-2) behaviour. Whilst essentially all possible species (Zn_1_-Zn_8_) are observed for the zinc form during the pH titration, only two metallospecies (Cd_7_ and Cd_4_) are dominating the speciation at different pH values. The abrupt transition from Cd_7_ to Cd_4_ below pH 3.9 is highly reminiscent of previous observations made for the cadmium-bound forms of mammalian MTs, where also pH-dependent cooperative loss of first three then four metal ions occurs [41,49]. This behaviour has been interpreted—e.g., for mammalian MTs, and in the previous study on S-tagged Cd-wMT-2 [39]—as the cooperative loss of a 3-metal cluster with lower pH stability than the remaining 4-metal cluster. The two-domain structure of mammalian MTs with an N-terminal M(II)_3_Cys_9_ and a C-terminal M(II)_4_Cys_11_ cluster is well established [50], and several other animal MTs are known to be two-domain proteins [3]. The concerted loss of three Cd(II) ions hence may indicate that the 20 Cys residues in wMT-2 probably also form a Cd_4_Cys_11_ and a Cd_3_Cys_9_ cluster in two separate domains. This hypothesis is also compatible with the sequence data shown in Figure 1; given the location of the gaps in the majority of sequences in the upper part of the alignment, and the high level of conservation of numbers and positions of Cys residues in the regions separated by these gaps, it seems highly probable that the region containing the first eleven Cys residues constitutes an N-terminal domain with a Cd_4_Cys_11_ cluster, and that the remaining nine Cys residues belong to a separate domain with a Cd_3_Cys_9_ cluster, with the less well conserved region between them constituting a linker between two domains. Further support for a two-domain structure comes from work on *Eisenia fetida* MT [38]; efforts to purify Cd-MT directly from worms led to the isolation of a 41-residue protein (*i.e.*, all residues before the gap in the alignment) harbouring four Cd(II) ions.

**Table 1:** Theoretical and observed masses for species detected during pH titrations of Cd- and Zn-wMT-2.

Metals Bound (*n*)	Neutral Masses for Cd_n_wMT-2		Neutral Masses for Zn_n_wMT-2	
	Observed	Theoretical	Observed	Theoretical
8	8678.0	8681.1	8303.5	8305.1
7	8570.5	8570.8	8240.0	8241.7
6	–	–	8175.0	8178.3
5	–	–	8112.0	8114.9
4	8239.9	8239.6	8050.5	8051.5
3	–	–	7984.0	7988.1
2	–	–	7916.0	7924.7
1	7908.5	7908.3	7856.5	7861.3
0 (apo)	7798.0	7797.9	7796.0	7797.9

Whilst conclusions regarding the two-domain structure of wMT-2, which have been reached previously already [39], are probably valid in the present case, we would caution that seemingly cooperative loss of metal ions may not necessarily correlate with loss of a cluster. An example to the contrary concerns a type 4 MT from plants, wheat E_C_, where the dominance of a Zn_4_ species does neither correlate with the loss of a Zn_2_ cluster nor the presence of a fully loaded Zn_4_ cluster or domain [44].

In contrast to the cadmium-bound form, species from Zn_8_ to Zn_5_ were observed for the zinc-bound protein, even at neutral pH. Over the past decade, it has become clear that the formation of “correctly” metallated species depends on the nature of the metal ions that are to be bound to the MT [51]. In general, mis-metallation and/or a broad speciation even at neutral pH (rather than one or two species as observed for Cd-wMT-2), are typical observations for MTs bound to “non-cognate” metal ions [51]. The low degree of cooperativity also testifies to a comparably low thermodynamic stabilisation of intact zinc-thiolate clusters, a notion that is also compatible with this view. Having noted this, it should be acknowledged that even MTs that are thought to be zinc-specific might not show much cooperativity towards zinc [52]; therefore, the degree of cooperativity in metal binding does not appear to be a suitable criterion to classify specificity *per se*.

Interestingly, the Zn_8_-species was not a major species at higher pH (see Figure 2f; pH 8.5), but became apparent only around neutral pH, together with larger quantities of under-metallated species. A closer look at the data for the Cd(II) form also reveals that once appreciable amounts of under-metallated (*i.e.*, Cd_4_) species are being formed, small amounts of a Cd_8_ species are also evident (Figure 3, pH 3.9 and 3.5). Considering the clean formation of Cd_7_ (Figure 2), the M_8_ species should be considered as an “over-metallated” species. Such over-metallated species have previously been observed, including for mammalian MTs [43,53]. In the present case, the existence of these species may indicate that these over-metallated species are more favourable than various possible under-metallated (and protonated) species, at least in the gas phase. The available data do not allow drawing conclusions regarding domain or cluster structures for these species, but in the case of mammalian MTs, it has been suggested that these “supermetallated” species may correspond to alternatively folded, single-domain structures [53].

Finally, the dominance of the Zn_4_ species observed at pH 4.2, and its subsequent rapid disappearance, may suggest a small degree of cooperativity in the predicted Zn_4_ cluster. This species predominated from pH 4.2–4.0, but at just 0.2 pH units lower, the likely Zn_4_ cluster collapsed, and the apo form dominated, with minor amounts of Zn_1_ and Zn_2_ present.

In summary, the data in Figure 2 and Figure 3 indicate considerable differences between the binding behaviour of wMT-2 towards Cd(II) and Zn(II). Whilst it needs to be acknowledged that in general, binding affinities of MTs for Zn(II) are several orders of magnitude lower than for Cd(II), this does not mean that the binding of Zn(II) is too weak for isolation and observation of fully metallated Zn-MTs —indeed, there are many examples to the contrary [43,51,54,55]. We hold that the observation of significant quantities of under- and over-metallated species at neutral pH or above is an indication for the fact that the Zn_7_ species is, overall, thermodynamically not much more favoured than the alternative species observed. To gain further insight into the origin of the observed differential behaviour, we next determined the average stability constants of the Zn(II) and Cd(II) complexes of wMT-2.

### 2.3. Affinities of wMT-2 for Cadmium and Zinc

**Figure 4:**
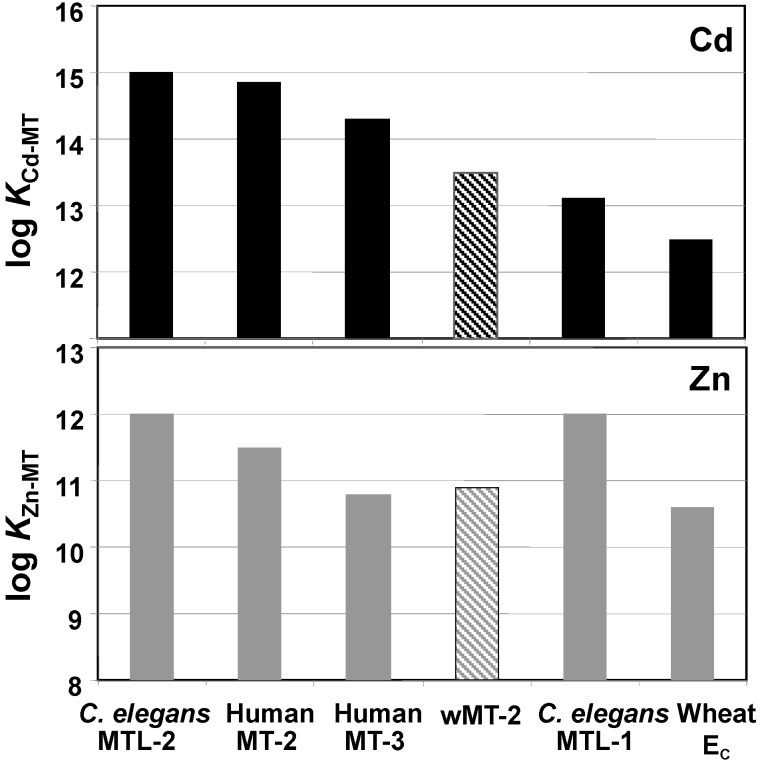
Comparison of stability constants determined by competition with 5F-BAPTA at pH 8.1 and *I* = 4 mM (10 mM Tris) [19,20,55,56]. The proteins are ordered by falling affinity for Cd(II). Hatched bars highlight wMT-2 data determined in the present study. Note that the scales do not start at 0.

Whilst competition with protons constitutes one possible measure of affinity and cluster stability that allows facile comparison between different MTs, it is desirable to also determine overall average stability constants. For both Zn- and Cd-wMT-2, this was achieved using a method based on competition with the chelator 5F-BAPTA (5-fluoro-1,2-bis(2-aminophenoxy)-ethane-*N*,*N*,*N′*,*N′*-tetraacetic acid) [56]. Figure 4 compares the values obtained (log *K*_Cd-MT_ = 13.5 ± 0.5 and log *K*_Zn-MT_ = 10.9 ± 0.5) with those determined for other MTs by the same method and under the same experimental conditions. It should be noted that these conditions include a relatively low ionic strength (*I* = 4 mM) and high pH (8.1), both factors that strengthen coordinative bonds in general, as low ionic strength renders charge recombination between metals and ligands more favourable, and high pH reduces the competition from protons for lone pairs.

The most salient point from the comparison shown in Figure 4 is that the affinity of wMT-2 for neither Zn(II) nor Cd(II) stands out. Its affinity for cadmium is not only lower than that of *C. elegans* MTL-2, another MT that is strongly induced by Cd(II) and with proven effects on Cd(II) homeostasis [19,20], but also lower than those measured for mammalian MTs, which are thought not to have evolved to serve in biological cadmium handling. Contrarily, its affinity for Zn(II) is even a little higher than that of the zinc-specific [57] wheat E*_C_*.

This comparison highlights that it is not the absolute affinity of an MT for a particular metal ion that is the most important parameter that relates to biological function, but the relative stabilities of the complexes with different metal ions, in a context of an ensemble of other proteins [1,19]. As long as the MT’s affinity for Cd(II) is higher than those of most other cellular proteins in *L. rubellus* (including those for handling zinc), and as long as the resulting complex has an appropriate lifetime that allows safe shuttling of the toxicant, wMT-2 will be able to effectively protect against toxic effects of cadmium. Furthermore, both pH-of-half-displacement values (Section 2.2) as well as competition reactions with a chelator (this section) indicate that overall affinities are not a major cause for the heterogeneity observed for the zinc-bound samples (Figure 2). Protein folds in the presence of Zn(II) and Cd(II) were studied next.

### 2.4. Folding Behaviour of Zn- and Cd-wMT-2

**Figure 5:**
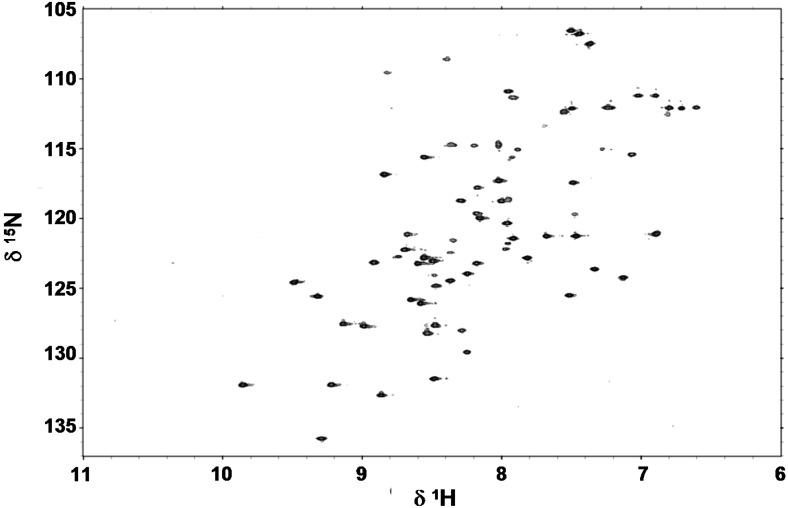
[^1^H,^15^N] HSQC spectrum (700 MHz) of ^13^C,^15^N-labelled Cd_7_wMT-2 (25 °C, 0.5 mM protein, 20 mM NH_4_HCO_3_, pH 6.8, 10% D_2_O). The broad dispersion of backbone N–H crosspeaks, and the narrow peak shapes indicate a well-folded protein.

**Figure 6:**
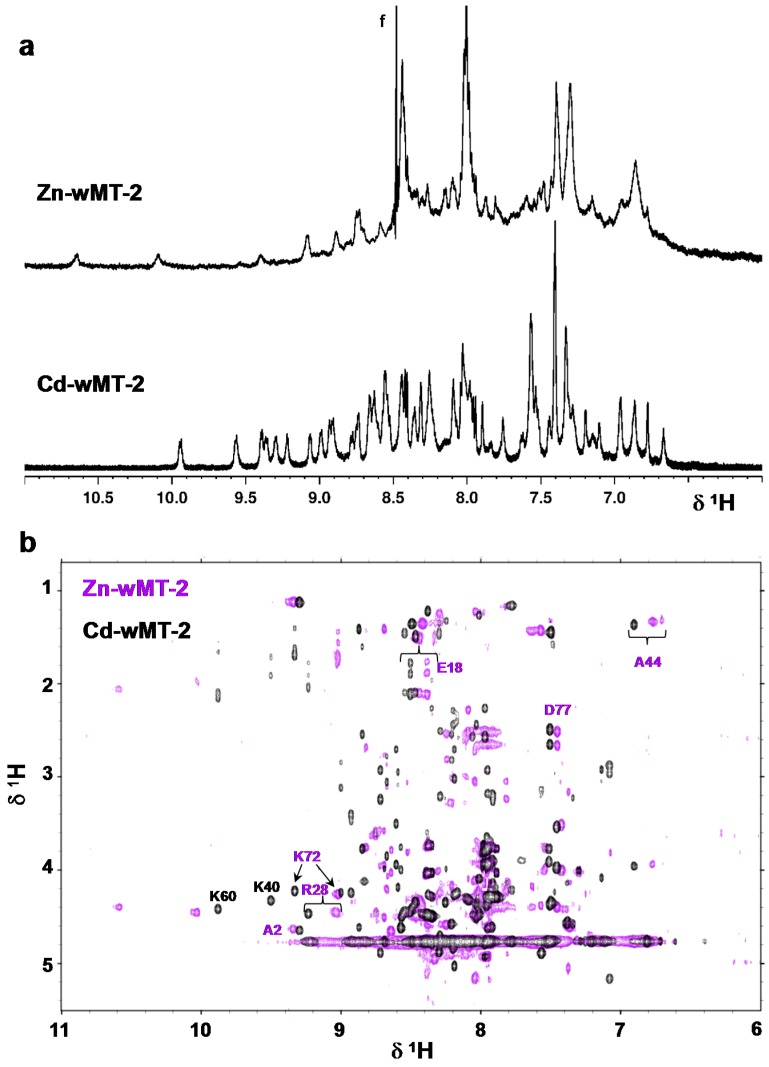
Comparison of 1D ^1^H (**a**) and 2D [^1^H,^1^H] TOCSY (**b**) NMR data for Zn- and Cd-wMT-2. Only the fingerprint regions (consisting mainly of N–H backbone resonances) are shown. Although all four spectra show considerable dispersion of N–H resonances, especially the comparison in (**a**) demonstrates that Zn-wMT-2 is considerably less well folded than Cd-wMT-2. Selected assigned residues are labelled in (**b**) for both proteins; labels in purple refer to residues observed for both forms; black labels refer to residues that were assignable for Cd-wMT-2 only. The “f” in (**a**) refers to the signal for formate at 8.48 ppm.

The folding behaviour of wMT-2 was studied by ^1^H NMR spectroscopy. Figure 5 shows a [^1^H,^15^N] HSQC spectrum of ^15^N,^13^C-labelled Cd_7_wMT-2. The dispersion and linewidths of the N–H crosspeaks indicate an overall well-folded protein. Figure 6a shows 1D ^1^H NMR spectra for Zn- and Cd-wMT-2. It is immediately evident that the number of resolved backbone N–H resonances is higher for Cd-wMT-2 than for Zn-wMT-2. The backbone N–H peaks for Zn-wMT-2 are generally broader, and there is a large amount of unresolved intensity in the random-coil region (around 8.0–8.4 ppm) for the Zn-loaded form. From these data, it is possible to conclude qualitatively that the Zn-loaded protein is much less well folded than the Cd-loaded protein. This is also borne out in the 2D [^1^H,^1^H] TOCSY NMR spectra for Zn- and Cd-wMT-2 compared in Figure 6b. Nevertheless, at least some ^1^H resonances for Zn-wMT-2 show considerable dispersion (see, e.g., the low-field-shifted peaks above 10.0 ppm), indicating that the Zn-loaded protein form is at least partially folded. Furthermore, and despite the heterogeneity in speciation as observed by mass spectrometry for Zn-wMT-2, the spectrum is dominated by just one species, as indicated by the almost complete absence of “split” peaks.

We have obtained a near-complete sequential assignment for Cd-wMT-2, and a partial assignment for Zn-wMT-2. Surprisingly, residues that could be assigned for Zn-wMT-2 are distributed throughout the entire sequence (a few of these are indicated in Figure 6b); therefore, we can exclude the possibility of just one of the two domains being folded in Zn-wMT-2. Thus, it appears that the Zn-bound protein is much more dynamic than its Cd-bound form, leading to significant portions of backbone ^1^H resonances not being resolvable or assignable.

Another conclusion that can be drawn from the 2D data in Figure 6b is that although some residues are in a similar environment in both forms (as indicated by the similarity in chemical shifts of their backbone N–H protons; see for example residues A2 and D77, both of which are close to the termini), there are also many residues with considerable (e.g., R28, K72), or even extremely large changes in chemical shift (e.g., the two unassigned, low-field shifted N-H resonances above 10 ppm for Zn-wMT-2). This is a strong indication for non-isostructural binding of Zn(II) and Cd(II). Typically, MTs, at least those for M(II) metal ions, are expected to bind these two metal ions in a structurally similar manner, although examples to the contrary are known, e.g., mammalian MT-3 [58], and plant type 4 MTs [57,59].

In conclusion, it is evident that the Cd-bound form is rather well and stably folded, whilst the Zn-bound form differs substantially from this form, both in terms of structure and dynamics.

## 3. Experimental Section

### 3.1. Materials

Unless stated otherwise, all chemicals and reagents were obtained from Fisher Scientific, Loughborough, UK. All solutions were prepared with purified water (MilliQ, Millipore, Nottingham, UK) and other reagents used were of analytical grade or better.

### 3.2. Protein Expression and Purification

*Lumbricus rubellus* MT-2 was recombinantly expressed in *E. coli* Rosetta 2(DE3)pLysS (Novagen, Nottingham, UK) cells as a fusion protein with an N-terminal S-peptide tag, using a pET29a-derived plasmid, with the *mt2* gene cloned between *Sal*I and *Nco*I restriction sites [31]. Cells were grown in standard Luria-Bertani medium at 37 °C (180 rpm), and Kanamycin (50 µg/mL) and Chloramphenicol (34 µg/mL) were used as selective antibiotics. Once an optical density at 600 nm of 0.6–0.8 was reached, protein over-expression was induced by 1 mM IPTG (Isopropyl-d-thiogalactopyranoside), and 0.5 mM ZnSO_4_ or 0.2 mM CdSO_4_ (both Sigma Aldrich, Dorset, UK) were added at the same time. Cells were harvested after 5–6 h of induction by centrifugation (5000× *g*, 10 min, 4 °C). Cell pellets were resuspended in 4–8 mL sonication buffer (50 mM Tris–HCl; 0.1 M KCl; 3 mM β-mercaptoethanol, 1% TWEEN-20, pH 8.5) per g wet cell weight. To prevent metal loss during sonication, 1 mM ZnSO_4_ or CdCl_2_ was added. The lysate was separated from cell debris by centrifugation for 30 min at 30,000× *g*, 4 °C. S-tagged wMT-2 was separated from the bulk of other contents of the cell lysate by gel filtration (GE Healthcare HiLoad 16/60, Superdex G75). After concentration to 1 mg/mL, the S-peptide tag was cleaved using bovine thrombin (50 units per mg protein; Sigma Aldrich, Dorset, UK) following manufacturer’s protocols, and the mixture was subjected to a second round of gel filtration. Protein concentrations were determined via measuring sulfur concentration by Inductively-Coupled Plasma Optical Emission spectroscopy (ICP-OES, Perkin Elmer, Seer Green, UK), or by determining thiol content via Ellman’s method [60], after removal of bound metal ions by incubation with EDTA (2,2′,2′′,2′′′-(Ethane-1,2-diyldinitrilo)tetraacetic acid, Sigma-Aldrich, Dorset, UK).

### 3.3. Determination of Protein Concentration and Metal-Protein Stoichiometries by ICP-OES

Samples of approximately 1 ppm [S] were prepared in 0.1 M ultrapure HNO_3_ (prepared in-house by sub-boiling point distillation; DuoPUR, Milestone S.R.L, Sorisole, Italy). Each sample was simultaneously assessed for Zn, Cd, Cu and S against mixed-element standards of concentrations between 0 and 2 ppm, which were prepared gravimetrically from high grade commercial stocks (1000 ppm, Fluka, Buchs, Switzerland). ICP-OES measurements were performed on an Optima 5300 DV instrument (Perkin Elmer, Seer Green, UK).

### 3.4. Mass Spectrometry

Samples were concentrated to 30–50 µM protein concentration using either Amicon Ultra centrifugal filtration units, with 3 kDa molecular weight cut-off (Fisher Scientific, Loughborough, UK), or Vivaspin centrifugal concentrators, with 5 kDa molecular weight cut-off (Sigma-Aldrich, Dorset, UK), using 20 mM ammonium bicarbonate (pH 8.35) buffer. Prior to mass spectrometric analysis, 10% (*v*/*v*) HPLC grade methanol was added to the samples, to obtain mass spectra of metallated species. The pH was gradually deceased using either acetic acid or formic acid. All samples were analysed on an HCTultra ion-trap mass spectrometer (Bruker Daltonics, Coventry, UK) equipped with an ESI source. Samples were directly infused using a syringe pump at 240 µL/h. Typically, data were acquired for 0.5–1.0 min in the positive mode over an *m*/*z* range of 500–3000. Resulting mass spectra were averaged, smoothed using the Savitzky-Golay algorithm, and where required deconvoluted onto a true mass scale using the Bruker Data Analysis Suite (Bruker Daltonics, Coventry, UK).

### 3.5. Affinity Measurements by ^19^F NMR Spectroscopy

Zn- and Cd-loaded forms of recombinant wMT-2 were buffer-exchanged into 10 mM Tris–HCl and 10% D_2_O, pH 8.1, using Amicon ultrafiltration devices (3 kDa molecular weight cut-off). Samples, approximately 450 µM in metal ion concentration, were incubated with 5-fluoro-1,2-bis(2-aminophenoxy)-ethane-*N*,*N*,*N′*,*N′*-tetraacetic acid (5F-BAPTA; 3 mM; Molecular Probes™, Invitrogen) overnight at 25 °C. Direct observe proton-decoupled 1D ^19^F NMR spectroscopy was carried out on a DRX400 spectrometer (Bruker) fitted with a QNP probe-head operating at 375.91 MHz for ^19^F. Datasets of 12,288 scans were acquired with a spectral width of 200 ppm, an acquisition time of 0.87 s and relaxation delay of 1.0 s. All spectra were acquired at 25 °C. FIDs were apodized with squared sine-bell functions, Fourier transformed with 64k data points and baseline corrected with TOPSPIN v. 2.1 software (Bruker Biospin, Coventry, UK). The apparent stability constants for metal-MT complexes were calculated using the method published by Hasler *et al.* [56]. The value for *K*_Cd(BAPTA)_ at 30 °C and *I* = 138 mM was corrected for temperature (25 °C) and ionic strength (*I =* 4 mM) used in our experiments to give a log *K* value of 11.75. The value for *K*_Zn(BAPTA)_ was also recalculated and a log *K* value of 9.91 was used. Metal and protein concentrations used in the calculations were determined accurately by ICP-OES.

### 3.6. ^1^H NMR Spectroscopy

Samples for ^1^H NMR spectroscopy were prepared in 20 mM NH_4_HCO_3_ buffer, pH 6.9, 10% D_2_O. All data were acquired for 0.5–1.0 mM samples on an AV II 700 (Bruker Biospin, Coventry, UK) equipped with a TCI cryoprobe.

Spectral conditions for TOCSY and NOESY experiments were: 25 °C; 48 scans; 4096 data points in F2 and 512 increments in F1, 90° pulse width ≈ 8.00 µs; spectral width 16.0 ppm in both F1 and F2. A mixing time of 60 ms was used for the TOCSY experiments; for NOESY experiments, several experiments with different mixing times were acquired (60–120 ms). Data were processed with TOPSPIN v. 2.1 (Bruker Biospin, Coventry, UK). Raw data were apodized using squared sine-bell functions and Fourier transformed with 2048 × 2048 data points, and visualised using SPARKY v.3.114 software [61].

## 4. Conclusions

We have studied the composition, metal affinity, and folding behaviour of recombinant *L. rubellus* wMT-2 in its Zn(II)- and Cd(II)-bound forms. As previously found for the S-tagged protein [39], wMT-2 binds seven Cd(II) ions optimally, with a high likelihood for the formation of two separate domains—an N-terminal domain harbouring a Cd(II)_4_Cys_11_ cluster and a C-terminal domain with a Cd(II)_3_Cys_9_ cluster. Two-domain MTs are common in animals: vertebrate, crustacean, sea urchin, and mussel MTs all have two domains, despite the absence of significant sequence similarity between these MTs [3]. At present, there is no clear opinion as to why such a two-domain arrangement may be advantageous—especially not in the case of MTs with a predominant role in detoxification. In MTs dealing with essential Zn(II) or Cu(I), two domains might serve in supporting interactions with different partners, and in some cases, the two domains might even handle different metal ions—a prime example is mammalian brain-specific MT-3 [62,63].

We have found significant differences in protein yields, metal stoichiometry, and folding behaviour between Zn-wMT-2 and Cd-wMT-2, which are congruent with the major biological function of wMT-2, which entails protection against Cd(II) toxicity [29]. The concept that MTs have evolved to optimally bind their cognate metal ion is a still emerging and hence debated concept. It is clear that metal selectivity and discrimination cannot be based on overall affinity [1], as all true MTs studied thus far show a similar order of binding affinity as small-molecule thiolate chelators, *i.e.*, Cu(I) > Cd(II) > Zn(II). This general trend, together with the knowledge that the protein backbones of MTs tend to be very flexible, may tempt one to regard MTs as not much more than larger versions of such small-molecule chelators, and to dismiss the relevance of protein folding. However, several recent studies on MTs from plants [44,55,59,64], snails [22,23,24,65,66,67], and nematodes [19,20,68] have demonstrated a correlation between the biologically “correct” metal ion and the “foldedness” of the respective MT. We consider that a correlation between cognate metal and protein folding also makes sense in the case of wMT-2, both in terms of fundamental (bio-)physical principles, as well as biological function: (i) even in cases where Zn(II) and Cd(II) are bound “isostructurally”, the difference in ionic radii (0.74 *vs.* 0.92 Å), and hence bond lengths, will require small but potentially significant adaptations in side-chain conformation and backbone fold, and therefore, differences in the energetics for fold stability depending on which metal is bound can be expected; (ii) In recognition of the fact that the “foldedness” of proteins impacts on their persistence *in vivo* [69], we expect that MTs synthesised in the presence of the “wrong” metal ion (in the present case Zn(II)) will have shorter *in vivo* lifetimes. This may help to remobilise the mis-incorporated metal ion, and prevent it from going down the wrong pathway.

These ideas are principally compatible with the finding that cadmium, but not other metal ions, are bio-accumulated in sulfur-rich vesicles by *L. rubellus*. As a next step, it would be desirable to test their validity by carrying out further metalloproteomic experiments in earthworms.
